# YAP1 enhances cell proliferation, migration, and invasion of gastric cancer *in vitro* and *in vivo*

**DOI:** 10.18632/oncotarget.13188

**Published:** 2016-11-07

**Authors:** Dan Sun, Xiaoting Li, Yingjian He, Wenhui Li, Ying Wang, Huan Wang, Shanshan Jiang, Yan Xin

**Affiliations:** ^1^ Laboratory of Gastrointestinal Onco-Pathology, Cancer Institute and General Surgery Institute, The First Affiliated Hospital of China Medical University, Shenyang 110001, Liaoning Province, China; ^2^ Key laboratory of Carcinogenesis and Translational Research (Ministry of Education), Peking University Cancer Hospital and Institute, Beijing, 100142, China

**Keywords:** yes-associated protein1/YAP1, gastric cancer, proliferation, migration, invasion

## Abstract

Yes-associated protein 1 (YAP1) plays an important role in the development of carcinomas such as breast, colorectal, and gastric (GC) cancers, but the role of YAP1 in GC has not been investigated comprehensively. The present study strongly suggests that YAP1 and P62 were significantly up-regulated in GC specimens, compared with normal gastric mucosa. In addition, the YAP1^high^ P62^high^ expression was independently associated with poor prognosis in GC (hazard ratio: 1.334, 95% confidence interval: 1.045–1.704, *P* = 0.021). Stable YAP1 silencing inhibited the proliferation, migration, and invasion of BGC-823 GC cells *in vitro* and inhibited the growth of xenograft tumor and hematogenous metastasis of BGC-823 GC cells *in vivo*. The mechanism was associated with inhibited extracellular signal-regulated kinases (ERK)1/2 phosphorylation, elevated E-cadherin protein expression and decreased vimentin protein expression, down-regulated β-catenin protein expression and elevated α-catenin protein expression, and down-regulated long non-coding RNA (lncRNA) expressions including HOX transcript antisense RNA (HOTAIR), H19, metastasis-associated lung adenocarcinoma transcript 1 (MALAT1), human large tumor suppressor-2 (LATS2)-AS1-001, and LATS2. YAP1 over-expression promoted the proliferation, migration, and invasion of human immortalized normal gastric mucosa GES-1 cells *in vitro* by reversing the above signal molecules. Subcutaneous inoculation of GES-1 cells and YAP1-over-expressing GES-1 cells into nude mice did not generate tumors. We successfully established the xenograft tumor models using MKN-45 GC cells, but immunochemistry showed that there was no YAP1 expression in MKN-45 cells. These results suggest that YAP1 is not a direct factor affecting tumor formation, but could accelerate tumor growth and metastasis. Collectively, this study highlights an important role for YAP1 as a promoter of GC growth and metastasis, and suggests that YAP1 could possibly be a potential treatment target for GC.

## INTRODUCTION

Gastric cancer is a leading cause of cancer-related death in China and most gastric cancer patients are already diagnosed with late stage disease [1]. Despite the best available therapeutic approaches, the 5-year survival of patients with GC ranges from 95% for stage IA to 10% for stage IIIC and 3% for stage IV [2]. Therefore, there is a need for the identification of novel targets for therapies.

The Hippo-YAP signaling pathway regulates cell proliferation and apoptosis [3]. Within this pathway, the Yes-associated protein 1 (YAP1) is a negative regulator of the Hippo-YAP pathway [4]. The WW domain of YAP1 directly interacts with the transcription factor polyomavirus enhancer binding protein 2α (PEBP2α) through the PPXY motif [4]. Therefore, YAP1 acts as a transcription co-activator of the Hippo-YAP signaling pathway together with PEBP2α [5]. In addition, YAP1 can co-activate other PPXY-motif-containing transcription factors such as receptor tyrosine-protein kinase erbB-4 (ERBB4) and p73 [6]. YAP1 interacts with the transcription factors TEA domain transcription factors (TEAD)1-4 and plays an essential role in mediating TEAD-dependent gene expression, which are involved in cell proliferation and survival [7].

On a tumorigenesis point of view, YAP1 promotes epithelial-mesenchymal transition (EMT), which is involved in cancer metastasis [8]. YAP1 over-expression in transgenic mice induced a dramatic increase of liver size and finally led to tumors [9]. Furthermore, YAP1 (located on 11q22) has been identified as a candidate oncogene in several cancers; its overexpression and increased nuclear localization have been reported in breast cancer [8], hepatocellular carcinoma [10], colorectal cancer [11], GC [12], pancreatic cancer [13], and esophageal squamous cell carcinoma [14]. A recent study showed that YAP1 overexpression was associated with the progression, lymph node metastasis, and poor prognosis of GC [15]. Another study showed that YAP1 was highly expressed in GC tissues compared with normal tissues from the same patients, but that YAP1 did not correlate with the clinicopathologic characteristics of the patients [16]. Therefore, there remain some controversies about the role of YAP1 in GC.

The purposes of the present study were to detect the expression of YAP1 in GC specimens, and correlation with the prognosis of patients with GC, to investigate the effects of stable YAP1 silencing and overexpression on the biological characteristics of GC and human immortalized normal gastric mucosa cells *in vitro* and *in vivo*, and to delineate the role of YAP1 in gastric tumorigenesis and progression.

## RESULTS

### YAP1 and P62 protein expressions in GC specimens and paired non-tumor gastric mucosa, and correlation with the prognosis of patients with GC

**Figure 1:**
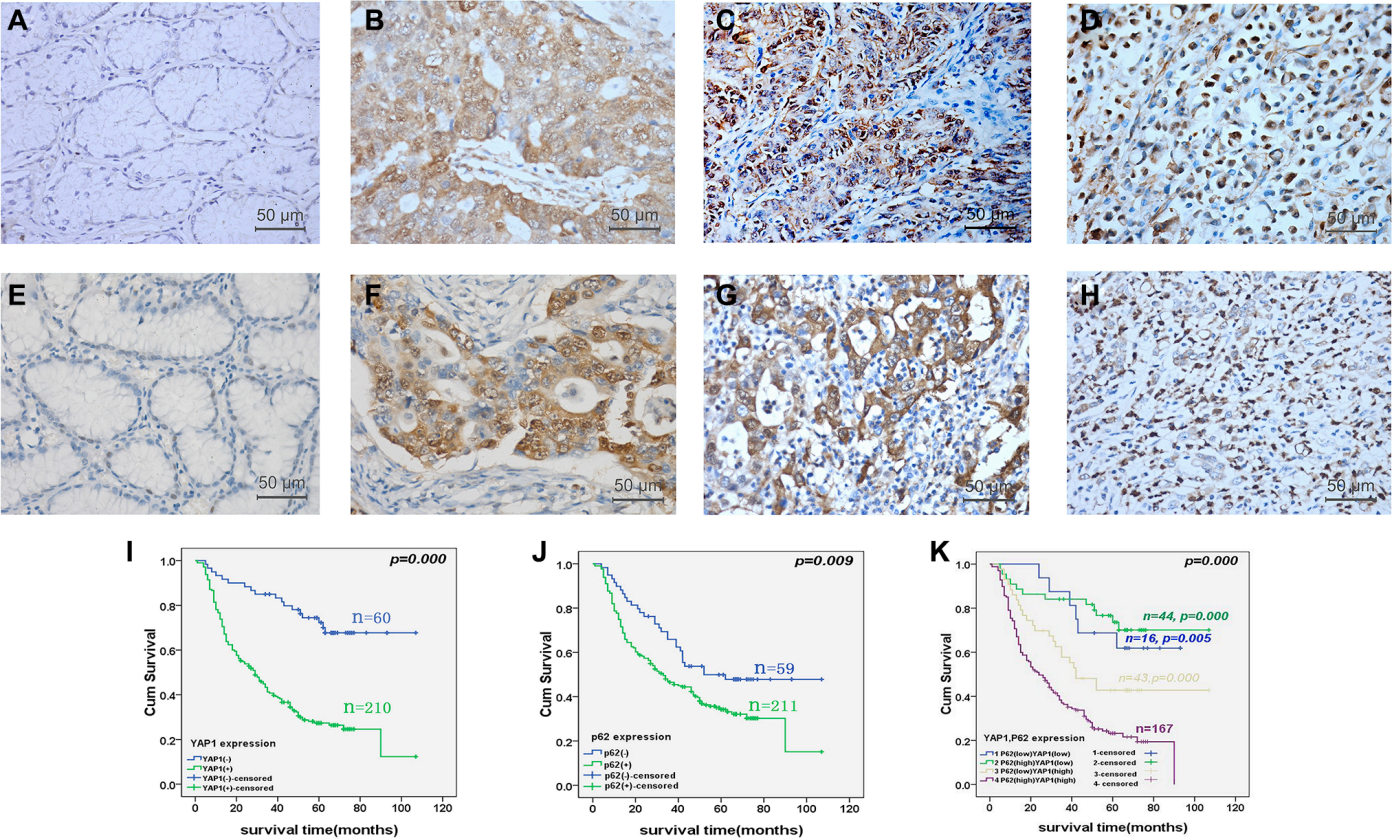
Yes-associated protein 1 (YAP1) and P62 protein expressions in gastric cancer (GC) specimens and paired non-tumor gastric mucosa, and correlation with the prognosis of patients with GC YAP1 and P62 protein expression was determined by immunohistochemistry (magnification: ×400). Compared with normal gastric mucosa (**A**), YAP1 expression was significantly up-regulated in moderately differentiated gastric adenocarcinoma (**B**), poorly differentiated adenocarcinoma (**C**), and signet ring cell cancer (**D**). Accordingly, compared with normal gastric mucosa (**E**), P62 expression was significantly up-regulated in moderately differentiated gastric adenocarcinoma (**F**), poorly differentiated adenocarcinoma (**G**), and signet ring cell cancer (**H**). Kaplan-Meier curves were plotted to determine the cumulative survival rate of patients with GC based on YAP1 and P62 protein expression, and showed that overall survival for patients with YAP1 high-expression was significantly worse than for those with low expression (*P* < 0.001) (**I**). Overall survival for patients with P62 high-expression was significantly worse than for those in the P62 low-expression group (*P* = 0.009) (**J**). Overall survival for patients with YAP1^high^P62^high^ indicated the worst prognosis, compare with other three groups (*P* = 0.005 *vs.* YAP1^high^P62^low^; *P* < 0.001 *vs.* YAP1^low^ P62^high^; *P* < 0.001 *vs.* YAP1^low^P62^low^) (**K**).

We assessed the YAP1 and P62 protein expressions by immunohistochemistry (IHC) in 302 GC specimens. YAP1 protein was located in the cytoplasm and nucleus. Compared with normal gastric mucosa, YAP1 expression was significantly up-regulated in moderately differentiated gastric adenocarcinoma, poorly differentiated adenocarcinoma, and signet ring cell cancer (Figure 1A–1D). Accordingly, compared with normal gastric mucosa, P62 expression was significantly up-regulated in moderately differentiated gastric adenocarcinoma, poorly differentiated adenocarcinoma, and signet ring cell cancer (Figure 1E–1H). YAP1 expression was high in 238 GC samples (78.8%, 238/302), which was significantly higher than in 64 GC samples with low expression (21.2%, 64/302) (*P* < 0.05). YAP1 expression was associated with Borrman's types (*P* = 0.041), WHO's histological types (*P* = 0.016), lymph node metastasis (*P* < 0.001), distant metastasis (*P* < 0.001), and TNM staging (*P* < 0.001), but was not associated with age, gender, Lauren's types, depth of invasion, and P62 expression (all *P* > 0.05) (Table 1).

**Table 1:** Characteristics of the patients with gastric cancer

Variable	*n*	YAP1 expression		HPR (%)	*P*
		Low	High		
**Age (year)**					0.927
≤ 55	110	23	87	79.1	
> 55	192	41	151	78.6	
**Gender**					0.387
Female	86	21	65	75.6	
Male	216	43	173	80.1	
**Borrmann's types**					**0.041**
I+II	46	15	31	67.4	
III+IV	255	49	206	80.8	
**WHO's histological types**					**0.016**
Papillary adenocarcinoma	4	0	4	100	
Tubular adenocarcinoma					
Well differentiated	16	8	8	50	
Moderately differentiated	67	16	51	76.1	
Poorly differentiated	174	37	137	78.7	
Undifferentiated carcinoma	4	0	4	100	
Signet ring cell adenocarcinoma	7	1	6	85.7	
Mucinous adenocarcinoma	30	2	28	93.3	
**Lauren's types**					0.792
Intestinal	92	18	74	80.4	
Diffuse	161	34	127	78.9	
Mixed	49	12	37	75.5	
**Depth of invasion**					0.078
T1+T2	49	15	34	69.4	
T3+T4	253	49	204	80.6	
**Lymph node metastasis**					**< 0.001**
N0	80	28	52	65	
N1-3	222	36	186	83.8	
**Distant metastasis**					**< 0.001**
M0	213	60	153	71.8	
M1	89	4	85	95.5	
**TNM staging**					**< 0.001**
I+II	112	39	73	65.2	
III+IV	190	25	165	86.8	
**P62 expression**					0.088
Low	66	19	47	71.2	
High	236	45	191	80.9	

YAP1: Yes-associated protein 1; HPR: High positive rate.

### YAP1^high^P62^high^ expression was independently associated with poor prognosis of GC

During the 107-month follow-up, 168 of 270 patients were known to be dead. The median survival was 51.8 ± 2.7 months. Kaplan-Meier curves showed that overall survival for patients with high-expression of YAP1 was significantly worse than for those with low expression (*P* < 0.001) (Figure 1I). Overall survival for patients with high-expression of P62 was significantly worse than for those in the low-expression group (*P* = 0.009) (Figure 1J). Overall survival for patients with YAP1^high^P62^high^ indicated the worst prognosis, compared with the other three groups (*P* = 0.005 *vs.* YAP1^high^P62^low^; *P* < 0.001 *vs.* YAP1^low^ P62^high^; *P* < 0.001 *vs.* YAP1^low^P62^low^) (Figure 1K). Furthermore, Kaplan-Meier analysis showed that Lauren's types, depth of invasion, lymph node metastasis, distant metastasis, and TNM staging were poor prognostic factors in GC (all *P* < 0.05) (Table 2).

**Table 2:** Univariate and multivariate analysis of prognostic factors in 270 patients with gastric cancer

Variable	Univariate analysis^a^			Multivariate analysis^b^		Multivariate analysis^c^	
	*n*	Mean survival (months)	*P*	HR (95% CI)	*P*	HR (95% CI)	*P*
**Borrmann's types**			0.698				
I+II	42	53.49					
III+IV	227	51.08					
**WHO's histological types**			0.810				
Papillary adenocarcinoma	4	38.75					
Tubular adenocarcinoma							
Well differentiated	14	36.14					
Moderate differentiated	57	54.68					
Poor differentiated	159	49.49					
Undifferentiated carcinoma	3	42.67					
Signet ring cell adenocarcinoma	5	48.20					
Mucinous adenocarcinoma	28	37.75					
**Lauren's types**			**0.032**	1.063 (0.846–1.336)	0.599	1.081 (0.861–1.358)	0.501
Intestinal	82	60.85					
Diffuse	142	44.40					
Mixed	46	44.57					
**Depth of invasion**			**< 0.001**	0.770 (0.403–1.471)	0.428	0.828 (0.432–1.588)	0.570
T1 + T2	44	79.72					
T3 + T4	226	46.53					
**Lymph node metastasis**			**< 0.001**	1.022 (0.859–1.216)	0.808	1.001 (0.842–1.188)	0.995
N0	74	75.80					
N1-3	196	41.99					
**Distant metastasis**			**< 0.001**	3.130 (1.483–6.607)	**0.003**	2.817 (1.328–5.978)	**0.007**
M0	184	70.49					
M1	86	12.01					
**TNM staging**			**< 0.001**	2.964 (1.741–5.044)	**< 0.001**	2.923 (1.711–4.995)	**<0.001**
I + II	101	85.68					
III + IV	169	31.85					
**YAP1 protein expression**			**< 0.001**	0.794 (0.563–1.121)	0.190		
Low	60	83.73					
High	210	41.90					
**P62 protein expression**			**0.009**	1.221 (0.813–1.833)	0.337		
Low	59	65.99					
High	211	46.63					
**YAP1 and P62 expression**			**< 0.001**			1.334 (1.045–1.704)	**0.021**
YAP1^low^P62^low^	16	72.87					
YAP1^low^P62^high^	44	84.69					
YAP1^high^P62^low^	43	60.27					
YAP1^high^P62^high^	167	36.27					
**Borrman and P62 expression**			**0.017**			1.004 (0.839–1.202)	0.964
Borrman I + II/p62^low^	25	51.83					
Borrman I + II/p62^high^	17	46.73					
Borrman III + IV/p62^low^	185	64.06					
Borrman III + IV/p62^high^	42	45.94					

a log rank test;

b Cox regression model (selected variables with *P*-values < 0.05 in the univariate analyses were considered for inclusion in the regression model. Did not include the combined variables (YAP1 and P62 expression, and Borrman and P62 expression)).

c Cox regression model (selected variables with *P*-values < 0.05 in the univariate analyses were considered for inclusion in the regression model. Did not include the individual variables (YAP1 protein expression, and P62 protein expression)).

HR: hazard ratio; 95%CI: 95% confidence interval.

The multivariate Cox proportional hazards regression model 1 (did not include the combined variables (YAP1 and P62 expression, and Borrman and P62 expression)) showed that distant metastasis (hazard ratio (HR): 3.130, 95% confidence interval (CI): 1.483–6.607, *P* = 0.003) and TNM staging (HR: 2.964, 95% CI: 1.741–5.044, *P* = 0.000) were independently associated with the prognosis of GC (Table 2). The multivariate Cox proportional hazards regression model 2 (did not include the individual variables (YAP1 protein expression and P62 protein expression)) showed that distant metastasis (HR: 2.817, 95% CI: 1.328–5.978, *P* = 0.007), TNM staging (HR: 2.923, 95% CI: 1.711–4.995, *P* < 0.001), YAP1 and P62 expression (HR: 1.334, 95%CI: 1.045–1.704, *P* = 0.021) were independent predictors of the prognosis of GC (Table 2).

### Effects of stable YAP1 silencing in BGC-823 cells and stable YAP1 overexpression in GES-1 cells on proliferation, clone formation ability, and cell cycle distribution *in vitro*

**Figure 2:**
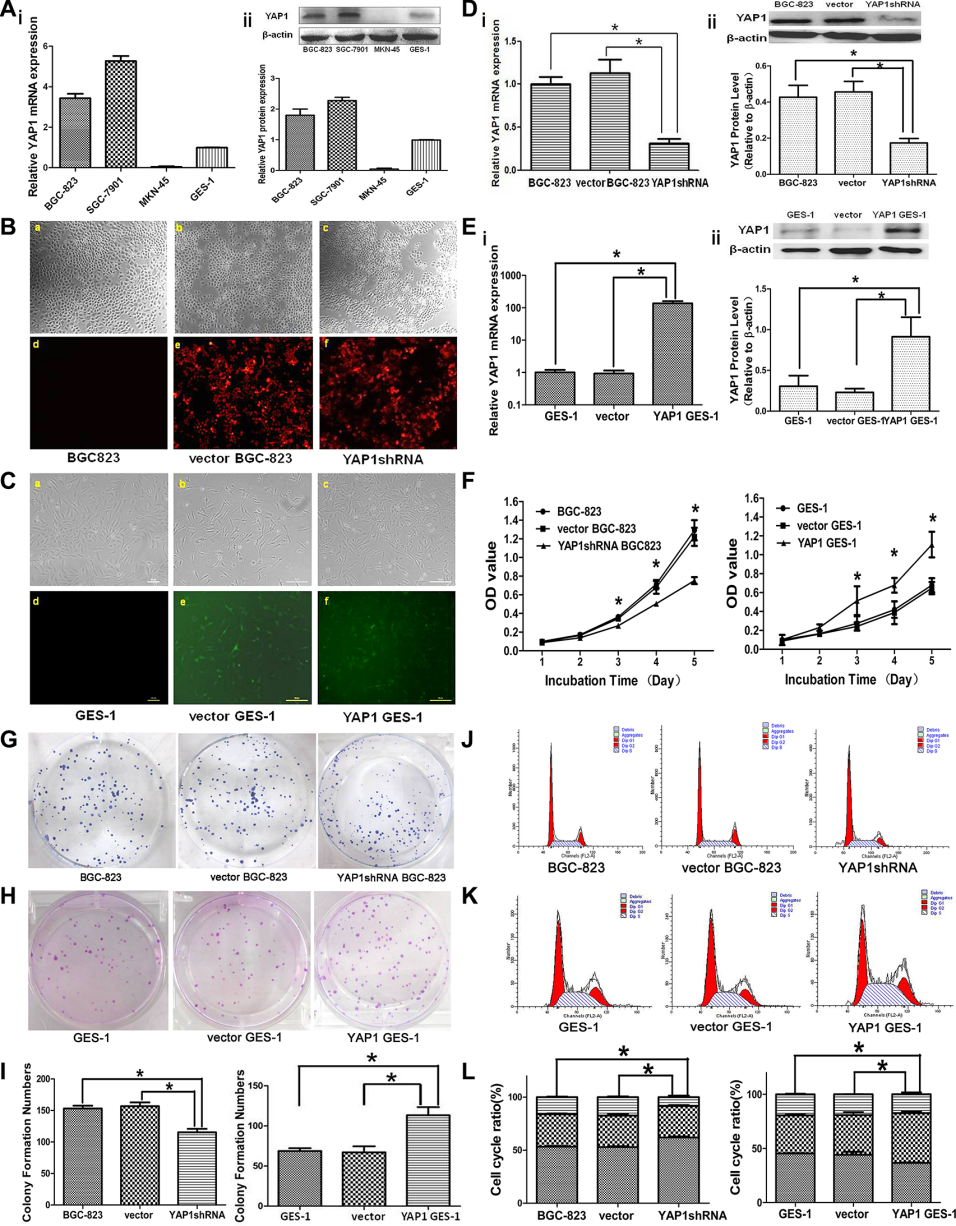
Effects of stable YAP1 silencing in BGC-823 cells and stable YAP1 overexpression in GES-1 cells on proliferation, clone formation ability, and cell cycle distribution *in vitro* (**A**) YAP1 mRNA (i) and protein (ii) expression levels in the human GC cell lines BGC823, MKN45, and SGC7901, and the human immortalized normal gastric mucosa cell GES-1, as determined by qRT-PCR and western blot, respectively. BGC-823: untreated BGC-823 cells; Vector BGC-823: BGC-823 cells stably transfected with pRFP-C-RS plasmid; YAP1 shRNA: BGC-823 cells stably transfected with pRFP-YAP1 shRNA; GES-1: untreated GES-1 cells; Vector GES-1: GES-1 cells stably transfected with pEGFP-C3; and YAP1 GES-1: GES-1 cells stably transfected with pEGFP-C3-YAP1 overexpression. (**B**) Expression of the red fluorescence protein (RFP) in the Vector BGC-823 (e) and YAP1 shRNA (f) groups under fluorescence microscope, but the RFP was not expressed in the BGC-823 group (d). (**C**) Expression of the green fluorescence protein (GFP) in vector GES-1 (e) and YAP1 GES-1 (f) groups under fluorescence microscope, but the GFP was not expressed in the GES-1 group (d). YAP1 mRNA (i) and protein (ii) expressions in BGC-823 (**D**) and GES-1 cells (**E**) as determined by qRT-PCR and western blot, respectively. (**F**) Cell proliferation in the BGC-823 and GES-1 cells was determined by MTT. Clone formation abilities in the BGC-823 cells (**G**, **I**) and GES-1 cells (**H**, **I**). Cell cycle distribution in the BGC-823 cells (**J**, **L**) and GES-1 cells (**K**, **L**) was determined by flow cytometry using propidium iodide (PI) staining. Data are shown as mean ± standard deviation (SD). *P < 0.05.

YAP1 mRNA and protein expressions were higher in BGC-823 and SGC-7901 GC cells compared with human immortalized normal gastric mucosa GES-1 cells, but there was no YAP1 expression in MKN-45 GC cells (Figure 2A).

Stable YAP1 silencing (YAP1 short hairpin RNA (shRNA)) in BGC823 cells and stable YAP1 overexpression in GES-1 cells were successfully established and validated by qRT-PCR and western blot. Expression of red fluorescence protein was observed in the vector BGC-823 group (BGC-823 cells stably transfected with pRFP-C-RS plasmid) and YAP1 shRNA BGC-823 group (BGC-823 cells stably transfected with pRFP-YAP1 shRNA), but not in the BGC-823 group (Figure 2B). Meanwhile, cells in the vector GES-1 group (GES-1 cells stably transfected with pEGFP-C3) and YAP1 GES-1 group (GES-1 cells stably transfected with pEGFP-C3-YAP1 overexpression) expressed green fluorescence protein (Figure 2C). There was an obvious inhibition of YAP1 mRNA and protein expressions in the YAP1 shRNA BGC-823 group compared with the BGC-823 and vector BGC-823 groups (Figure 2D), and the mRNA and protein expressions of YAP1 in the YAP1 GES-1 group was higher than in the GES-1 and vector GES-1 groups (Figure 2E).

To understand whether YAP1 modulated proliferation and colony formation of GC and normal gastric mucosa cells, we performed 3-(4,5-dimethylthiazol-2-yl)-2,5-diphenyltetrazolium bromide (MTT) and colony formation assays. As shown in Figure 2F, optical density (OD) values at 490 nm of the YAP1 shRNA BGC-823 group (0.27 ± 0.02, 0.51 ± 0.01, 0.75 ± 0.04) were significantly lower than those in the vector BGC-823 (0.35 ± 0.03, 0.68 ± 0.07, 1.22 ± 0.09) and BGC-823 (0.36 ± 0.03, 0.71 ± 0.04, 1.29 ± 0.11) groups (all *P* < 0.05) from day 3 to day 5. Meanwhile, OD values at 490 nm of the YAP1 GES-1 group (0.51 ± 0.15, 0.68 ± 0.08, 1.11 ± 0.14) were significantly higher than those in the vector GES-1 (0.24 ± 0.04, 0.39 ± 0.12, 0.65 ± 0.06) and GES-1 (0.27 ± 0.08, 0.42 ± 0.09, 0.68 ± 0.08) groups (all *P* < 0.05) from day 3 to day 5.

The colony formation assay showed that the colonies in the YAP1 shRNA BGC-823 group were smaller and fewer than those formed in the vector BGC-823 and BGC-823 groups (Figure 2G). The number of colonies in the YAP1 shRNA BGC-823 group was reduced (both *P* < 0.05, Figure 2I), but the GES-1 cells transfected with YAP1 overexpression showed increased colony formation compared with the GES-1 and vector GES-1 groups (both *P* < 0.05, Figure 2H and 2I).

Flow cytometry (FCM) using propidium iodide (PI) staining was used to access whether YAP1 modulated cell cycle distribution. Knockdown of YAP1 resulted in G0/G1 cell cycle arrest (BGC-823: 53.3 ± 1.4%; vector BGC-823: 52.9 ± 1.5%; YAP1 shRNA BGC823: 62.1 ± 1.6%; both *P* < 0.05) and reduction of S phase cells in BGC-823 cells (Figure 2J and 2L). Accordingly, the percentages of cell in G0/G1 phase of YAP1 GES-1 group (36.6 ± 0.7%) was significantly lower than that of the GES-1 (45.4 ± 0.8%) and vector GES-1 (44.2 ± 4.8%) groups (both *P* < 0.05, Figure 2K and 2L).

### Effects of stable YAP1 silencing in BGC-823 cells and stable YAP1 overexpressing in GES-1 cells on migration and invasion abilities *in vitro*

**Figure 3:**
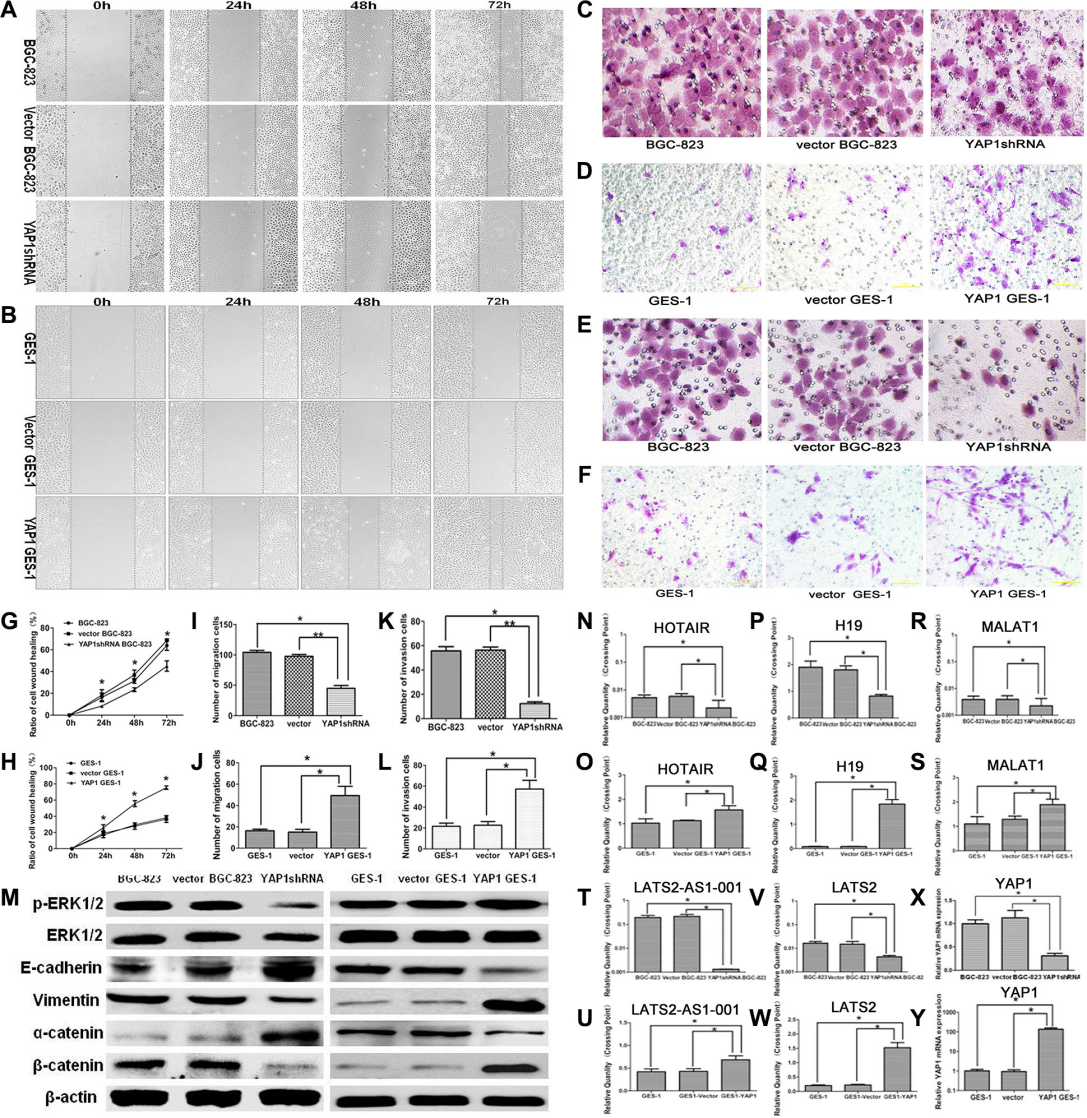
Effects of stable YAP1 silencing in BGC-823 cells and stable YAP1 overexpressing in GES-1 cells on migration and invasion abilities *in vitro* The wound healing assay was used to evaluate the migration properties of BGC-823 GC cells (**A**, **G**) and GES-1 cells (**B**, **H**). Cells were photographed 0, 24, 48, and 72 h after wounding (magnification×100). The Transwell assay was used to evaluate the migration properties of BGC-823 GC cells (**C**, **I**) and GES-1 cells (**D**, **J**) (magnification: ×200). The Transwell assay was used to evaluate the invasion properties of BGC-823 GC cells (**E**, **K**) and GES-1 cells (**F**, **L**). (**M**) p-ERK1/2, ERK1/2, epithelial-related protein E-cadherin, mesenchymal related protein Vimentin, α-catenin, and β-catenin expressions were determined by western blot. β-actin was used as an inner control. HOX transcript antisense RNA (HOTAIR) (**N**, **O**), H19 (**P**, **Q**), metastasis-associated lung adenocarcinoma transcript 1 (MALAT1) (**R**, **S**), human large tumor suppressor 2 (LATS2)-AS1-001 (**T**, **U**), and LATS2 (**V**, **W**) LncRNA expressions and YAP1 mRNA expression (**X**, **Y**) in BGC-823 and GES-1 cells were determined by qRT-PCR. Data are shown as mean ± SD. *P < 0.05; **P < 0.01.

Wound-healing assay, Transwell migration, and invasion assay were performed to study the effect of YAP1 on the migration and invasion abilities of GC cells and normal gastric mucosa cells. As shown in Figure 3A and 3G, the wound healing rate of the YAP1 shRNA BGC-823 group (8.3 ± 0.5%, 23.4 ± 1.7%, 45.0 ± 4.8%) was decreased compared with that of the BGC-823 (16.3 ± 4.0%, 31.4 ± 2.2%, 63.9 ± 4.5%) and vector BGC-823 (18.3 ± 5.3%, 36.9 ± 4.4%, 68.4 ± 1.6%) groups at 24, 48, and 72 h (all *P* < 0.05). On the other hand, the wound healing rate of the YAP1 GES1 group (31.6 ± 6.5%, 66.1 ± 5.0%, 87.0 ± 2.4%) was increased compared with that of the vector GES-1 (14.8 ± 2.5%, 27.9 ± 4.0%, 36.5 ± 4.1%) and GES-1 (16.9 ± 3.4%, 29.5 ± 1.7%, 38.2 ± 2.7%) groups at 24, 48, and 72 h (all *P* < 0.05, Figure 3B and 3H).

The Transwell migration assay confirmed the results of the wound healing assay. After 24 h of incubation, knockdown of YAP1 led to a reduction of BGC823 cell migration capacity (Figure 3C and 3I), and YAP1 over-expression promoted cell migration ability of GES-1 cells (Figure 3D and 3J). The numbers of cells invading the Matrigel filter in the YAP1 shRNA BGC-823 group were less than that of the vector BGC-823 and BGC-823 groups (both *P* < 0.05, Figure 3E and 3K). On the other hand, compared with the GES-1 and vector GES-1 groups, the number of cells invading the Matrigel filter in the YAP1 GES-1 group was significantly increased (both *P* < 0.05, Figure 3F and 3L).

### Effects of stable YAP1 silencing in BGC-823 cells and stable YAP1 overexpressing in GES-1 cells on the protein expressions of p-ERK1/2, ERK1/2, E-cadherin, vimentin, α-catenin, and β-catenin, and LncRNA expressions including HOTAIR, H19, MALAT1, LATS2-AS1-001, and LATS2

Western blotting showed that knockdown of YAP1 in the YAP1 shRNA BGC-823 group suppressed the protein expression of p-ERK1/2 compared with the BGC-823 and vector BGC-823 groups (Figure 3M). EMT was inhibited, as shown by elevated E-cadherin protein expression and decreased vimentin protein expression in the YAP1 shRNA BGC-823 group compared with the BGC-823 and vector BGC-823 groups (Figure 3M). Compared with the BGC-823 and vector BGC-823 groups, β-catenin protein expression in the YAP1 shRNA BGC-823 group was significantly down-regulated, while the expression of α-catenin was obviously elevated (Figure 3M). In GES-1 cells, YAP1 overexpression significantly up-regulated the protein expression of p-ERK1/2, vimentin, and β-catenin, and down-regulated the expression of E-cadherin and α-catenin (Figure 3M). It is well known that the expression of long non-coding RNA (LncRNA) plays critical roles in tumorigenesis. qRT-PCR showed that knockdown of YAP1 in the BGC-823 cells displayed down-regulated expressions of HOX transcript antisense RNA (HOTAIR), H19, metastasis-associated lung adenocarcinoma transcript 1 (MALAT1), human large tumor suppressor 2 (LATS2)-AS1-001, and LATS2 compared with the BGC-823 and vector BGC-823 groups (all *P* < 0.05, Figure 3N, 3P, 3R, 3T, 3V and 3X). Accordingly, the expressions of HOTAIR, H19, MALAT1, LATS2-AS1-001 and LATS2 were elevated in GES-1 cells overexpressing YAP1 compared with the GES-1 and vector GES-1 groups (all *P* < 0.05, Figures 3O, 3Q, 3S, 3U, 3W and 3Y).

### Stable knockdown of YAP1 inhibited xenograft tumor growth and lung metastasis, but YAP1 was not related with tumorigenesis *in vivo*

**Figure 4:**
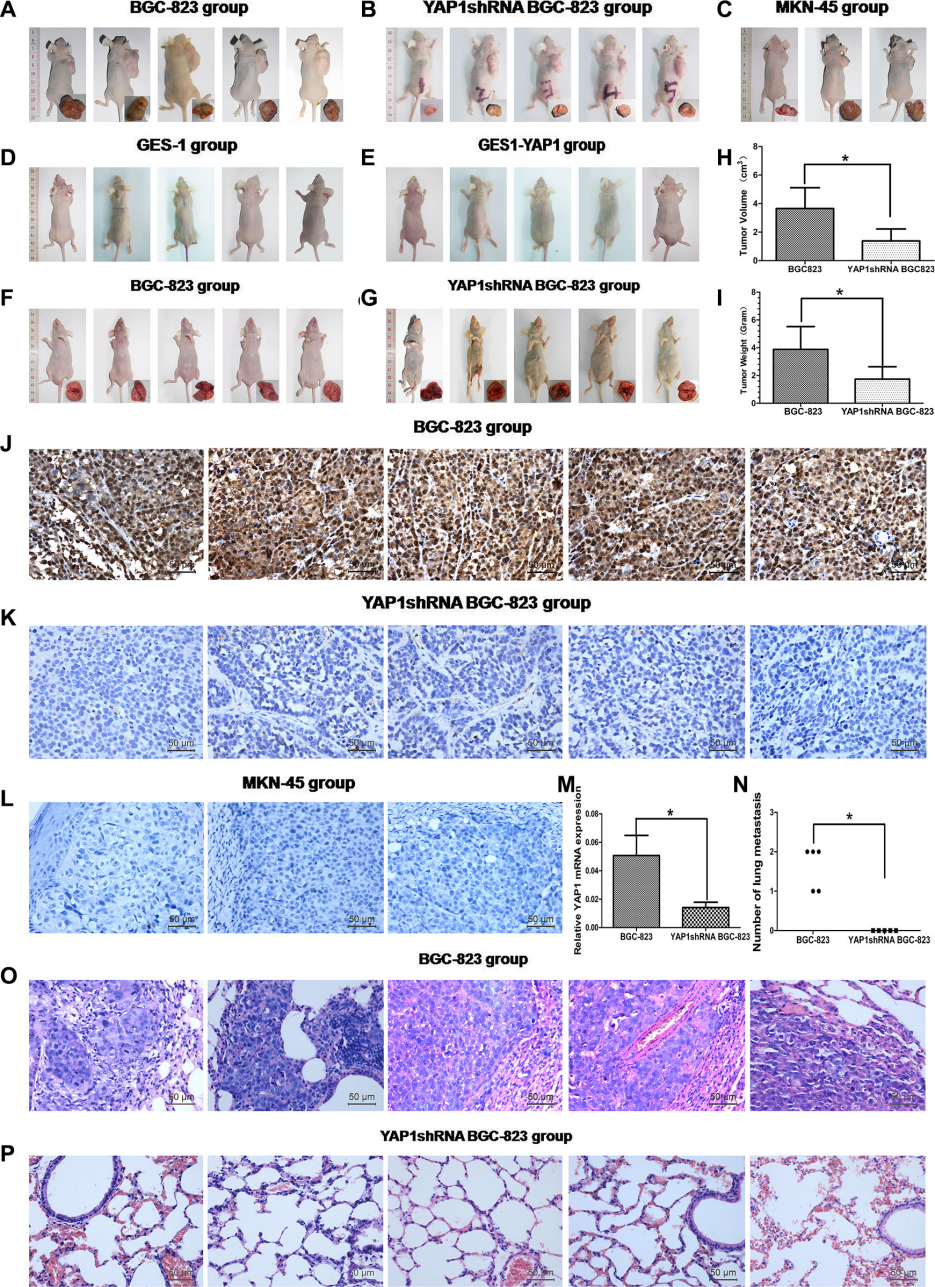
Stable knockdown of YAP1 inhibited xenograft tumor growth and lung metastasis, but YAP1 was not related with tumorigenesis *in vivo* BALB/c-nude mice were injected subcutaneously into the right flanks with BGC-823 (**A**), YAP1-shRNA BGC-823 (**B**), MKN45 (**C**), GES-1 (**D**) and GES-1-YAP1 (**E**) cells, and let to grow for 4 weeks to establish the heterotopic xenograft tumor mice model. (**H**) Tumor volume and (**I**) tumor weight in BGC-823 and YAP1 shRNA BGC-823 groups. YAP1 protein expression was detected by immunohistochemistry (magnification: ×400) in BGC-823 (**J**), YAP1-shRNA BGC-823 (**K**) and MKN45 (**L**) groups. (**M**) YAP1 mRNA levels in BGC-823 and YAP1 shRNA BGC-823 groups were detected by qRT-PCR. Lung tissues and H&E staining of lung sections (magnification: ×400) in the BALB/c-nude mice hematogenous metastasis models that were harvested from the mice that had been injected in the lateral tail veins with BGC-823 (**F**, **O**) and YAP1 shRNA BGC-823 cells (**G**, **P**), 8 weeks after inoculation. (**N**) The numbers of metastatic foci per section of lung of individual mouse was measured after 8 weeks. *P < 0.05.

BALB/c-nude mice were injected subcutaneously into the right flanks with BGC-823, YAP1-shRNA BGC-823, MKN45, GES-1, and GES-1-YAP1 cells, and let to grow for 4 weeks to establish the heterotopic xenograft tumor mice model. Results showed that silencing YAP1 significantly inhibited tumor growth. The tumor volume and tumor weight in the YAP1 shRNA BGC-823 group was smaller than those in the BGC-823 group (both *P* < 0.05, Figure 4A, 4B, 4H and 4I). IHC showed that the YAP1 protein expression in the YAP1 shRNA BGC-823 group was lower than that in the BGC-823 group (Figure 4J and 4K). YAP1 mRNA levels in the YAP1 shRNA BGC-823 group was significantly lower than that in the BGC-823 group (*P* < 0.05, Figure 4M). All three mice in the MKN-45 group displayed tumors 28 days after cell inoculation (Figure 4C), but IHC showed that MKN-45 cells did not express YAP1 (Figure 4L). Mice injected subcutaneously with GES-1 cells and GES-1 cells overexpressing YAP1 did not generate tumors (Figure 4D and 4E). BGC-823 or YAP1shRNA BGC-823 cells were injected into nude mice through the tail vein. Stable knockdown of YAP1 reduced the number of lung metastases (*P* < 0.05, Figure 4F, 4G, 4O, 4P, and 4N).

## DISCUSSION

YAP1 is an effector of the Hippo pathway, which promoted cell proliferation and tumor growth in mammals [17, 18], but the influence of YAP1 in GC has not been comprehensively investigated. In the present study, the expression of YAP1 protein in GC tissues was elevated, which is supported by previous studies [15, 16]. Moreover, high-expression of YAP1 was associated with Borrman's types, WHO's histological types, lymph node metastasis, distant metastasis, and TNM staging. Hu et al. demonstrated that YAP1 overexpression was associated with progression, lymph node metastasis, and poor prognosis of GC, suggesting that overexpression of YAP1 could be a predictor of lymph node metastasis [15].

P62, encoded by proto-oncogene c-myc, is required for tumor transformation; P62 is overexpressed in several types of cancer [19, 20]. Qian et al. and Su et al. showed that P62 expression was common in gastrointestinal tract carcinomas and associated with cell differentiation and tumor metastasis [21, 22]. In the present study, the multivariate COX regression analysis revealed that the YAP1^high^ P62^high^ expression pattern was independently associated with poor prognosis of patients with GC.

To investigate the effect of YAP1 on the behavior of GC, we silenced the expression of YAP1 in BGC-823 cells and overexpressed it in GES-1 cells. Down-regulating YAP1 expression reduced the proliferation and colony formation ability of cells by suppressing the phosphorylation of ERK1/2, increasing the α-catenin expression, and arrested the cells in G0/G1 phase. Inactive ERK1/2 is mostly located in the cytoplasm and transfers into the nucleus once activated to induce the transcription of cancer genes such as c-fos and c-myc [21, 22]. The sustained activation of ERK1/2 eventually promotes cell proliferation and malignant transformation. Elevated phosphorylated c-Raf/MEK1/2/ERK1/2 was observed in MKN45 cells stably expressing YAP1, and YAP1 expression could activate ERK1/2 signaling and resulted in c-fos induction [12]. Huo et al. demonstrated that the inhibition of YAP1 expression sensitized HCC cells to doxorubicin by decreasing the levels of phosphorylated ERK1/2 [23]. Furthermore, a previous study demonstrated that α-catenin is a tumor suppressor that inhibits YAP1 activity and that YAP 1 is a key driver of keratinocyte proliferation induced by α-catenin loss [24]. YAP1 acts downstream of α-catenin to control epidermal proliferation [3]. α-catenin depletion or deletion in keratinocytes relocalizes YAP1 from the cytoplasm to the nucleus [25]. Taken together with these previous studies, our results suggest that silencing YAP1 increase the expression of α-catenin possibly by a negative feedback effect. In addition, the cell cycle results of the present study are consistent with those observed in different cancers (clear cell renal cell carcinoma [26], osteosarcoma [27], and GC [12]). It has been reported that accumulation of G1 cells was increased in MKN1 and AGS GC cells in which YAP1 expression was silenced [12]. Further analyses in 786-0 cells in which YAP1 is down-regulated demonstrated cell cycle G0/G1 arrest and inhibited cell proliferation [26].

Knockdown of YAP1 expression in SGC7901, MKN1, and AGS cells inhibited cell migration and invasion [12]. In the present study, the wound healing and Transwell assays showed that YAP1 could promote the cell migration and invasion abilities of GC *in vitro*. These effects could be attributed to promote EMT as shown by decreased epithelial-related protein E-cadherin expression and increased mesenchymal-related protein vimentin expression when YAP1 is overexpressed; on the other hand, using YAP1 shRNA, EMT was inhibited, as shown by elevated E-cadherin protein expression and decreased vimentin protein expression. Inducing EMT is a key feature of YAP signaling in mammalian tumor cells [28, 29], except in head and neck squamous cell carcinoma (HNSCC) [30] and non-small cell lung cancer (NSCLC) [31]. Zhang et al. found that over-expression of YAP1 in MCF10A cells resulted in the down-regulation of E-cadherin and concomitant up-regulation of N-cadherin and fibronectin [16, 28]. Loss of E-cadherin often induces the up-regulation of the β-catenin pathway [32] and the transcriptional activity of β-catenin is closely related to EMT [33, 34]. Combined β-catenin and YAP1 silencing impairs the growth of human hepatoblastoma cells, while constitutively activated β-catenin and YAP1 trigger liver tumor development in mice [35]. Moreover, β-catenin is the cross point between Wnt and Hippo signaling pathways. YAP1 hinders β-catenin translocation to the nucleus and eventually suppresses Wnt signaling pathway [36, 37]. Consistent with these previous studies, our results showed that silencing YAP1 reduced the expression of β-catenin.

LncRNAs play important roles in tumorigenesis. HOTAIR is up-regulated in lung cancer, breast cancer, esophageal cancer, and GC [38]. HOTAIR is involved in the control of cell apoptosis, growth, metastasis, angiogenesis, DNA repair, and tumor cell metabolism [38]. Patients with high expression of HOTAIR have a poor prognosis [38]. In the present study, YAP1 overexpression increased the expression of HOTAIR. Up-regulation of H19 contributes to poor prognosis in patients with GC [39]. It was reported that YAP1 and H19 expression levels were elevated in bladder cancer cells, and H19 expression was found to be significantly associated with YAP1 expression [40]. In addition, YAP1 was found to enhance H19 expression, whereas H19 had no significant effect on YAP1 expression in bladder cancer [40]. Consistently, the present study found that YAP1 overexpression increased the expression of H19. MALAT1 is overexpressed in lung cancer, GC, and cervical cancer [41]. YAP1 up-regulates MALAT1 expression in liver cancer, whereas serine/arginine-rich splicing factor 1 (SRSF1) played an opposing role [41]. Consistently, we also observed that YAP1 overexpression increased the expression of MALAT1.

LATS2 plays a critical role in Hippo signaling [42]. YAP1 activation results in activation of their negative regulators, LATS1/2 kinases, to constitute a negative feedback loop of the Hippo pathway in both cultured cells and mouse tissues. YAP1 in complex with the transcription factor TEAD directly induce LATS2 expression. YAP1 also stimulate the kinase activity of LATS1/2 through inducing neurofibromin 2 (NF2) [43]. LATS2-AS1-001, a pseudogene transcript of LATS2 antisense RNA1 (LATS2-AS1) has not been reported. In the present study, YAP1 overexpression significantly increased LATS1/2 and LATS2-AS1-001 expressions, suggesting that there was a negative feedback loop of the Hippo pathway.

Kang et al. showed that xenograft tumor growth induced by YAP1-expressing GC cells was significantly enhanced compared with control cells [12]. Similar results were shown in oral squamous cell carcinoma [44], human gallbladder cancer [45], osteosarcoma [27], esophageal cancer [46], cervical cancer [47], and ovarian cancer [48]. However, the present study suggests for the first time that YAP1 overexpression could promote tumor growth but do not affect tumorigenesis. Indeed, down-regulation of YAP1 in BGC-823 GC cells obviously reduced tumor growth *in vivo*. In addition, MKN-45 GC cells induced tumor formation despite having no YAP1 expression [12]. These evidences suggest that YAP1 expression is not a direct factor affecting tumor formation, but that YAP1 over-expression could accelerate tumor cell growth. Song et al. suggested that YAP1 was able to confer cancer stem cell properties onto a wide variety of non-transformed cell types of gastrointestinal origin, including primary isolated esophageal epithelium cells, immortalized embryonic liver cells, and esophageal cancer cells [46]. In the present study, GES-1 cells (human immortalized normal gastric mucosa cells) were selected to overexpress YAP1 to examine whether YAP1 has an effect of tumoriginesis. The results showed that both GES-1 and YAP1-overexpressing GES-1 cells inoculated into mice could not form tumor, suggesting that the YAP1 gene might not involve in tumorigenesis. So far, this finding has not been reported.

Finally, the present study is the first to report that silencing YAP1 in BGC-823 GC cells significantly suppressed hematogenous metastatic spread of tumor cells. The tail vein injection model is known to have some disadvantages. Indeed, the cells do not follow the biological steps of metastasis formation [49] and ignore the crosstalk between the primary and metastatic tumors [50]. Although we agree that the tail vein injection model is not perfect to mimic the metastasis of cancer, it could nevertheless provide some clues about the metastatic and invasive abilities of cancer cells, when the cells are considered as having detached themselves from the primary tumor [51]. A recent study showed that the tail vein injection model was equivalent to the orthotopic injection model [52]. Nevertheless, additional study is necessary to further address the potential role of YAP1 in migration/invasion *in vivo*, since there is a possibility that reduced tumor expansion in the lung was due to YAP1 role in inhibiting the cell cycle that contributed to the observation.

In summary, elevated expression of YAP1 was observed in GC and was associated with the progression and metastasis of GC, and YAP1^high^ P62^high^ expression pattern were independently associated with poor prognosis of patients with GC. Furthermore, YAP1 effectively promoted the proliferation, colony formation, migration, and invasion of BGC-823 GC cells and human immortalized normal gastric mucosa GES-1 cells *in vitro.* The mechanism was associated with EMT, ERK1/2, α-catenin, and β-catenin, and with lncRNAs including HOTAIR, H19, MALAT1, LATS2-AS1-001, and LATS2. In addition, stable knockdown of YAP1 inhibited xenograft tumor growth and lung metastasis, but YAP1 was not related to tumorigenesis *in vivo*. Taken together, these findings suggest that YAP1 could be a potential target for GC therapy.

## MATERIALS AND METHODS

### Clinical specimens

Surgically resected GC specimens (*n* = 302) and paired non-tumor gastric mucosa (PNTG) (collected > 5 cm away from the edge of the primary tumor) were collected from August 2007 to October 2012 at the First Affiliated Hospital of China Medical University. None of the patients had received chemotherapy or radiotherapy before surgery. The characteristics of the patients are shown in Table 1. The study was approved by the Clinical Research Ethics Committee of the First Affiliated Hospital of China Medical University. Written informed consent was obtained from all study participants.

### Immunohistochemistry

Tissue microarrays of GC and gastric mucosa were constructed, and then cut to 4-μm sections. IHC for YAP1 and P62 was performed according to the manufacturer's instructions using rabbit anti-human YAP1 polyclonal antibody (1:100; Cell signaling; #4912) and mouse anti-human p62 polyclonal antibody (1:500; MBL; M162-3). Phosphate-buffered saline (PBS) was used as negative control.

YAP1 or P62 positivity was defined as the clear presence of brown granules in cytoplasm or nuclei and was assessed by two independent experienced pathologists blinded to the characteristics of the patients. The score was made according to the proportion of positive cells (0, none; 1, ≤10 %; 2, 11–25 %; 3, 26–50 %; 4, > 50 %). The intensity score was assigned for the average intensity of positive cells (0, none; 1, weak; 2, intermediate; 3, strong). The final score was the product of the two subscores, ranging 0–12. The expression was categorized as negative (score 0), (−); low (score 1–3), (1+); intermediate (score 4–6), (2+); and high (score 7–12), (3+). Patients were classified into two groups: scores 0–1+ were considered as low expression, while 2+**−**3+ were considered as high expression.

### Cell culture

The human GC cell lines BGC823, MKN45, and SGC7901, and the human immortalized normal gastric mucosa cell GES-1 were obtained from the Cancer Research Institution of China Medical University (China). The cells were cultured in RPMI-1640 (Hyclone, Logan, USA) supplemented with 10% fetal bovine serum (FBS) (Hyclone, Logan, USA) and 1% penicillin-streptomycin (Solarbio, Beijing, China), and incubated in 5% CO_2_ at 37°C.

### YAP1 silencing/overexpressing in cell lines

After preliminary experiment (two sequences were identified as specific YAP1-shRNA targeted sequences), the pRFP-YAP1 shRNA targeting sequence of human YAP1 gene (Genbank No. NM_001130145.2) (5′-GAT Ccc aga gaa tca gtc aga gaT TCA AGA Gat ctc tga ctg att ctc tgg TTT TTT A-3′) was chosen for the subsequent experiments. The non-coding plasmid pRFP-C-RS (Origene, USA) was used as a negative control. The plasmids for pEGFP-C3 and pEGFP C3-YAP1 overexpression were purchased from Addgene Co. The pRFP-YAP1 shRNA and pRFP-C-RS plasmids were transfected into BGC-823 GC cells. The pEGFP-C3 and pEGFP-C3-YAP1 plasmids were transfected into GES-1 cells. All plasmids were transfected into cells using Lipo-fectamine™ 2000 (Invitrogen, Carlsbad, USA), according to manufacturer's instructions.

Stable YAP1 shRNA BGC-823 and negative control cells (Vector BGC-823) were selected by puromycin. Stable YAP1 GES-1 and negative control cells (Vector GES-1) were selected by G418. All clones were picked out by fluorescence microscopy (Nikon eclipse Ti-s, Nikon Digital sight DS-U3) and cultured for at least 6 weeks before confirming the expression of YAP1 by qRT-PCR and western blotting.

### Quantitative real-time reverse transcriptase -PCR

Total RNA was isolated using the EASYspin Plus kit (Aidlab Biotechnologies, Beijing, China), according to the manufacturer's instructions. Isolated RNA was reverse-transcribed to cDNA with the PrimeScript^®^ RT reagent kit (TaKaRa, Dalian, China). Primer sequences were: YAP1 sense, 5′-TAC GAT ACA AGG CTG TTA GAG AG-3′ and anti-sense, 5′-TTG AGA TGC ATG CTT TGC ATA C-3′; GAPDH sense, 5′-GAA GGT GAA GGT CGG AGT C-3′ and antisense, 5′-GAA GAT GGT GAT GGG ATT TC-3′; HOTAIR sense, 5′-ggT AgA AAA AgC AAC CAC gAA gC-3′ and anti-sense, 5′-ACA TAA ACC TCT gTC TgT gAg TgC C-3′; H19 sense, 5′-ACT CAg gAA TCg gCT CTg gAA-3′ and anti-sense, 5′-CTg CTg TTC CgA Tgg TgT CTT-3′; MALAT1 sense, 5′-TAg gAA gAC AgC AgC AgA CA gg-3′ and anti-sense 5′-TTg CTC gCT TgC TCC TCA gT-3′; LATS2-AS1-001 sense, 5′-CTC TGG CAC TCC TAC T-3′ and anti-sense, 5′-CTG GAC CTG AAC CTA C-3′; and LATS2 sense, 5′-AGC TGG ACT CTG TGA AGC TG-3′ and anti-sense 5′-TGT CCA CCT TAC AAG CAA GG-3′. The PCR reaction was performed in 10 μl, including each primer, diluted cDNA templates, and SYBR^®^ Premix TaqTM II (TaKaRa, Dalian, China). The conditions were: 1) 95°C for 30 s; and 2) 40 cycles of 95°C for 5 s, annealing at 58.5°C for 30 s; dissolving curve at 95°C for 15 s, 60°C for 30 s, and 95°C for 15 s. The data was calculated using the 2^−ΔΔCt^ method, normalized to GAPDH. Each cDNA sample was run in triplicate.

### Western blotting

Cells were lyzed with a lysis buffer containing phenylmethyl sulfonylfluoride (PMSF) (Beyotime, Shanghai, China) at 4°C. Proteins were quantified using a BCA protein assay kit (ComWin Biotech, Beijing, China). Protein lysates (50 μg) were separated by 10% SDS-PAGE gels (Invitrogen) and transferred onto a polyvinylidene difluoride (PVDF) membrane (Beyotime, Shanghai, China). The membranes were blocked with 5% non-fat dry milk in Tris-phosphate buffer containing 0.05% Tween 20 (TBS-T) for 1 h at room temperature and then treated with the primary antibodies at 4°C overnight. After washing with TBST, the membranes were incubated with peroxidase-conjugated affinipure goat anti-rabbit IgG (H+L) (ZSGB-BIO, 1:5000, ZB-2301) and peroxidase-conjugated affinipure goat anti-mouse IgG (H + L) (ZSGB-BIO, 1:5000, ZB-2305) for 1 h at room temperature. The blots were visualized using an ECL kit (ComWin Biotech, Beijing, China), and quantified using the Image J Software, normalized to β-actin. The primary antibodies were: E-cadherin (1:500, Cell signaling, #3195), Vimentin (1:1000, Cell signaling, #5741), ERK1/2 (1:1000, Cell signaling; #4695), p-ERK1/2 (Thr202/Tyr204) (1:1000, Cell signaling, #9101), α-catenin (1:500, Proteintech, Catalog number: 66221-1-Ig), β-catenin (1:1000, Santa Cruz, sc-7963), and β-actin (1:1000, Bioss).

### Cell proliferation and colony formation assays

Cells were plated in 96-well plates at 2 × 10^3^ cells per well. MTT assays were performed every day over the following 5 days. Cells were incubated for 4 h in 20 μL MTT (Sigma, USA) (0.5 mg/ml) at 37°C. The color was developed by incubating the cells in 150 μL dimethylsulfoxide (DMSO, Sigma) and the plates were gently shaken for 10 min. The absorbance was detected at 490 nm.

For the colony formation assay, cells were cultured in six-well plates at 200 cells/well. After 12 days, the cells were fixed in 4% paraformaldehyde and stained with Giemsa (Sigma, USA). Colonies were then photographed under a Nikon eclipse Ti-S microscope (Nikon, Japan) and scored.

### Cell cycle

Cells were washed twice with cold PBS, fixed in 70% ethanol at 4°C for 24 h, and stained with 0.05 mg/ml of PI (Beyotime, Shanghai, China) and 0.05 mg/ml of RNase A (Beyotime, Shanghai, China) for 30 min in the dark. The cells were analyzed using a FACSCalibur (BD, USA).

### Wound healing assay

Cells were seeded into 6-well plates at 3 × 10^5^ cells/well. When reached 90% confluence, a single wound was created in the center of the well with a 10-μl sterile plastic tip. After washing the well with PBS, the cells migrating into the wounded areas were visualized and photographed at 0, 24, 48, and 72 h. The cell wound healing rate was calculated to assess cell mobility.

### Transwell migration and invasion assays

The cell migration assay was performed using a 24-well Transwell polycarbonate filters (8-μm pore size, Corning, USA). The cells were trypsinized and added to the upper chamber in 200 μl of serum-free medium containing 2 × 10^5^ cells, while the lower chamber contained 500 μl of RPMI-1640 supplemented with 20% fetal FBS. After incubating for 24 h, the cells passing through the filter were fixed with 4% paraformaldehyde, stained with 1% crystal violet, and counted under a Nikon eclipse Ti-S microscope.

For the cell invasion assay, the Transwell chambers were coated with 200 μl of Matrigel at a dilution of 1:7 in serum-free medium and the incubation time was extended to 48 h. The remaining of the methods was same as for the cell migration assay.

### Heterotopic xenograft tumor model and hematogenous metastasis model

Four-week old female BALB/c-nude mice were purchased from Beijing Vital River Laboratory Animal Co., Ltd., China. All animals were maintained under a sterile environment at the Animal Laboratory Unit of the China Medical University. The animals were grouped: BGC-823, BGC-823 stably transfected with pRFP-YAP1 shRNA, MKN45 (which do not express YAP1) [12], GES-1, and GES-1 cells stably transfected with pEGFP C3-YAP1 overexpression. After resuspension in PBS, 5 × 10^6^ cells in 200 μL were injected subcutaneously into the right flanks of the nude mice. Tumor volume was measured weekly using a digital caliper according to the formula: TV (mm^3^) = length × width^2^ × 0.5. All the mice were sacrificed 4 weeks after cell inoculation. Tumors were excised, photographed, measured, and weighted. IHC and qRT-PCR was used to assess the RNAi efficiency *in vivo*.

For the hematogenous metastasis model, BGC823 cells and BGC823 cells stably transfected with pRFP-YAP1 shRNA were collected in the logarithmic growth phase and inoculated into the tail vein at 5 × 10^6^/100 μL. Eight weeks later, the mice were sacrificed. H&E staining was used to detect lung metastasis.

All procedures and animal experiments were approved by the Animal Care and Use Committee of the First Affiliated Hospital of China Medical University.

### Statistical analysis

Statistical analysis was performed using SPSS 17.0 (IBM, Armonk, NY, USA). Categorical data were presented using proportion and compared with the chi-square test or the Fisher's exact test, as appropriate. All quantitative data were expressed as mean ± standard deviation and analyzed using the Student's *t*-test or one-way ANOVA with Bonferroni as post hoc test, as appropriate. The Kaplan-Meier and log-rank tests were used for survival analysis. Multivariable analysis model was run using a Cox proportional hazards regression model (enter method). All statistical analyses were two-sided, and significance was assigned at α = 0.05.
